# Supplementary material for: Deep learning with self-supervision and uncertainty regularization to count fish in underwater images
Source: PLoS One. 2022 May 4;17(5):e0267759. doi: 10.1371/journal.pone.0267759 (PMC9067705; doi:10.1371/journal.pone.0267759)
Supplement: Attachment — Submitted filename: Comments.docx [file pone.0267759.s003.docx]

**Comments:**

Page 2

- This is not true, several studies have shown that cameras can attract or scare some species of fish, that can be consider an intrusion in the sense that it disrupts the behavior and natural dynamics of fish populations. No method is non-intrusive, or non-biased. Check refs: Bacheler, *Comparing relative abundance, lengths, and habitat of temperate reef fishes using simultaneous underwater visual census, video, and trap sampling*, 2017; Campbell, *Comparison of relative abundance indices calculated from two methods of generating video count data*, 2015, for an idea on the biases of several methods, including cameras.

Page 3

- This is not entirely true. Softwares like Echoview or ESP3 can process sonar images in bulk, and they also can count fish in them. Echoview has the option to add user developed scripts in case several files from different sources are needed (so it would be fully-automated).
  I agree with the assertion that there are not many products, readily available, that process sonar images in a fully-automated way, but that depends on the definition of fully-automated. A well-written script in Echoview or ESP3 can do that.
  I could not find a full copy of [10] to corroborate that they had a similar conclusion.
  I don't think the advantage of a computer vision model is that it can automatically process vast numbers of images, but that it takes them and processes them as a whole.

Page 5

- Was there any criterium for the selection of this amount of images?

Page 8

Is this a hypotesis?

- Do you have preliminary data to support this?

Page 9

- What kind of information?

Page 14

- This classification may not be significant since the size of a dolphin is much greater than the one of a mullet so it is easily differentiated. The uncertainty introduced by the nets is more significant since the size of the "blobs" are comparable to the size of the mullets. This begs the questions: in the presences of other targets of similar size, does the CNN have a good performance too?
  Is the CNN considering past entries, or the way fish move to make its predictions?
  Human observers can tell if it is a net or a fish because they have access to past and future entries (frames) and can recognize the difference in the way that a net and a fish move. Given only still images, humans would have a much harder time recognizing the blobs associated with nets.

Page 15

- Although an interesting exercise, I don't see how this relates to the counting on sonar images.
  These are two fundamentally different CNNs, especially their datasets. Underwater images (DeepFish) are visual data, the frequencies (colors) are directly associated with a bandwidth in the environment. Sonar images' gray scales are due to the mapping of the device, the data is sound mapped into a visual space, and thus, their bandwidth is dependent on the design of the device and the processing of the data.
  I am not sure that you can compare both.
  It is interesting to see that the framework does better that the original DeepFish architecture, but that does not necessarily means it would be better in sonar images.

Page 17

- Is this speculation?

Page 18

- See my comment on the "Evaluation of DeepFish dataset". The better performance of the framework here does not necessarily means a better performance with sonar images. The two datasets are fundamentally different.
- I agree that ARIS acoustic images can be treated as low-resolution images. However, they are not the same as low-resolution visual images. They might be closer to low-resolution monochromatic images.

Page 19

- It is hard to find a study that does not support this. Only if the dataset is large enough, augmenting it does not help.
- But this was not the goal of the study
- Was there an attempt to quantify this?
- There are models (such mean count and maximum count) used by marine biologist that work with visual data that could be applied here to give an estimate of the error in the Ground Truth, that, as the authors suggest, will be compounded into the CNN
